# Oligodendrocyte ablation triggers central pain independently of innate or adaptive immune responses in mice

**DOI:** 10.1038/ncomms6472

**Published:** 2014-12-01

**Authors:** Simon Gritsch, Jianning Lu, Sebastian Thilemann, Simone Wörtge, Wiebke Möbius, Julia Bruttger, Khalad Karram, Torben Ruhwedel, Michaela Blanfeld, Daniel Vardeh, Ari Waisman, Klaus-Armin Nave, Rohini Kuner

**Affiliations:** 1 Institute for Pharmacology, University of Heidelberg, Im Neuenheimer Feld 366, 69120 Heidelberg, Germany; 2 Institute for Molecular Medicine, University Medical Center of the Johannes Gutenberg University of Mainz, 55131 Mainz, Germany; 3 Department of Neurogenetics, Max Planck Institute of Experimental Medicine, 37075 Goettingen, Germany; 4 Center Nanoscale Microscopy and Molecular Physiology of the Brain (CNMPB), Göttingen, Germany

## Abstract

Mechanisms underlying central neuropathic pain are poorly understood. Although glial dysfunction has been functionally linked with neuropathic pain, very little is known about modulation of pain by oligodendrocytes. Here we report that genetic ablation of oligodendrocytes rapidly triggers a pattern of sensory changes that closely resemble central neuropathic pain, which are manifest before overt demyelination. Primary oligodendrocyte loss is not associated with autoreactive T- and B-cell infiltration in the spinal cord and neither activation of microglia nor reactive astrogliosis contribute functionally to central pain evoked by ablation of oligodendrocytes. Instead, light and electron microscopic analyses reveal axonal pathology in the spinal dorsal horn and spinothalamic tract concurrent with the induction and maintenance of nociceptive hypersensitivity. These data reveal a role for oligodendrocytes in modulating pain and suggest that perturbation of oligodendrocyte functions that maintain axonal integrity can lead to central neuropathic pain independent of immune contributions.

## Abstract (web summary)

Whether oligodendrocytes have a role in the development of chronic pain is not clear. Here the authors show that oligodendrocyte depletion causes a neuropathic pain that sets in before demyelination and is independent of immune cell activation and infiltration.

## Graphical abstract

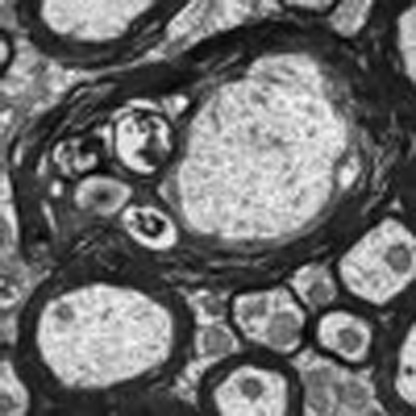

Chronic pain is a major clinical problem and underlying mechanisms are elusive. In contrast to the more classically studied forms of chronic inflammatory and peripheral neuropathic pain disorders, very little research has been conducted into mechanisms of neuropathic pain triggered by lesions or disorders of the central nervous system (CNS).

Although glia have been classically believed to solely nurture neurons, the last years have ushered in a new awareness of their importance in a variety of neural functions and disorders. Converging evidence indicates a key role for glia and neuro-glia interactions in the development and maintenance of neuropathic pain^1^,^2^,^3^,^4^. Three types of glial cells have been at the centre of investigation, namely microglia and astrocytes in the CNS, and satellite glial cells in the dorsal root ganglia (DRG) and trigeminal ganglia^1^,^2^,^3^,^4^. It has been postulated that ‘gliopathy’, a state involving dysfunctional glia, is a key contributor in cellular mechanisms contributing to neuropathic pain^2^,^4^.

Studies on chronic pain, however, have not addressed another key glial cell type, which is abundant in the CNS, namely, oligodendrocytes that generate myelin for axonal insulation in the CNS. However, recent studies have shown additional roles for oligodendrocytes in supporting axonal functions and long-term integrity, which may be independent of myelin^5^, and hold significance for neurological and psychiatric disorders^5^. However, whether oligodendrocyte function or pathology is linked with pain has not been addressed so far.

Here we use a genetic strategy employing diphtheria toxin (DTX)-diphtheria toxin receptor (DTR) system^6^ to specifically ablate oligodendrocytes cell-intrinsically in mice. Previous studies using this strategy to ablate oligodendrocytes have conclusively shown that primary oligodendrocyte loss leads to secondary axonal damage in the brain independent of CNS inflammation^7^,^8^,^9^,^10^,^11^. Here we show that primary oligodendrocyte ablation leads to a nociceptive hypersensitivity phenotype that closely resembles central neuropathic pain. The data suggest that a loss of oligodendrocyte functions that maintain axonal integrity in the spinal cord can trigger neuropathic pain and thus present a viable addition to immune dysregulation as a target for therapeutic interventions.

## Results

### Oligodendrocyte ablation in adult mice affects nociception

**Figure 1:**
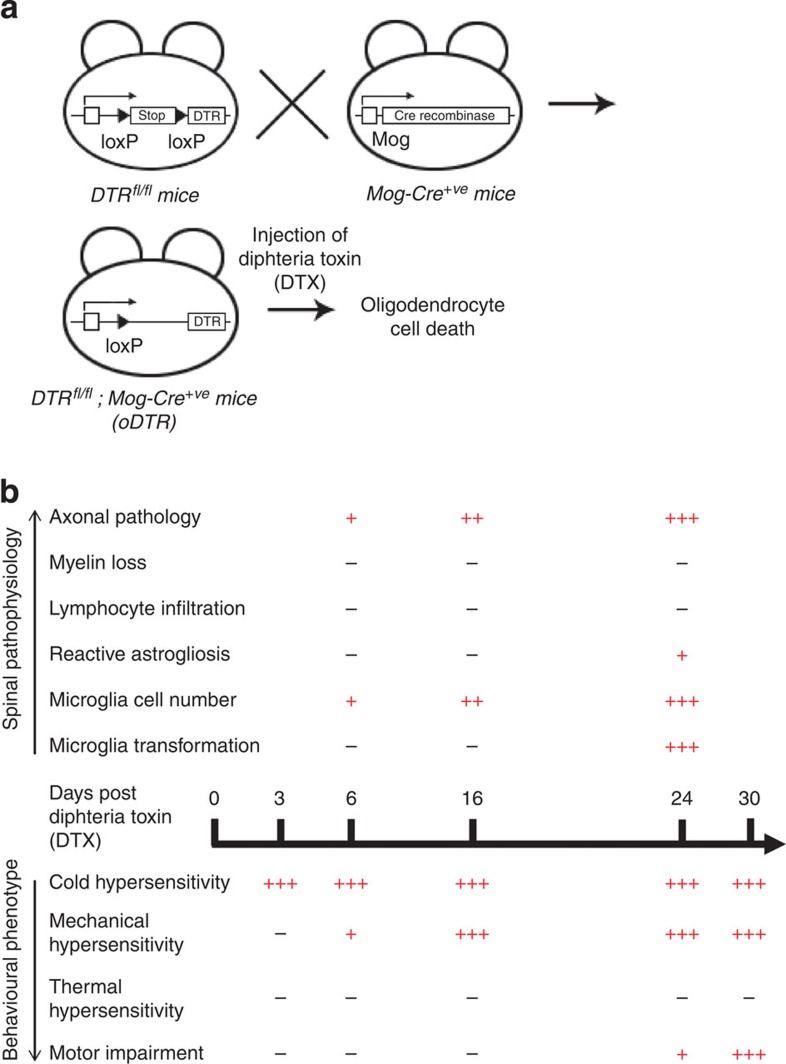
Scheme of inducible ablation of oligodendrocytes in adult mice and an overview of the resulting spinal pathophysiology and behavioural phenotypes. (**a**) Generation of mice harbouring a floxed stop cassette before a diphtheria toxin receptor (DTR) allele and Cre-recombinase specifically expressed in oligodendrocytes and induction of oligodendrocyte ablation following systemic injection of diphtheria toxin (DTX). (**b**) Absence (−) or presence (graded+signs depending upon severity) of various pathophysiological features in the spinal cord of mice up to 30 days following DTX administration and a concurrent analysis of sensitivity to noxious and non-noxious stimuli and motor impairment.

We achieved inducible ablation of oligodendrocytes in adult mice by mating mice expressing the DTR in a conditional locus, which enables expression of DTR in a cre recombinase-dependent manner (DTR^fl/fl^ mice; Fig. 1a)^6^, with mice expressing cre recombinase specifically in oligodendrocytes (*Mog-Cre*^*+ve*^ mice; Fig. 1a). Double transgenic mice (*DTR*^*fl/fl;*^*Mog-Cre*^*+ve*^—referred to as oDTR henceforth) were then treated with DTX systemically in adult stages (at least 8 weeks of age) to induce oligodendrocyte cell death. *Mog-Cre*^*+ve*^ mice and *DTR*^*fl/fl*^ mice treated with DTX served as controls.

**Figure 2:**
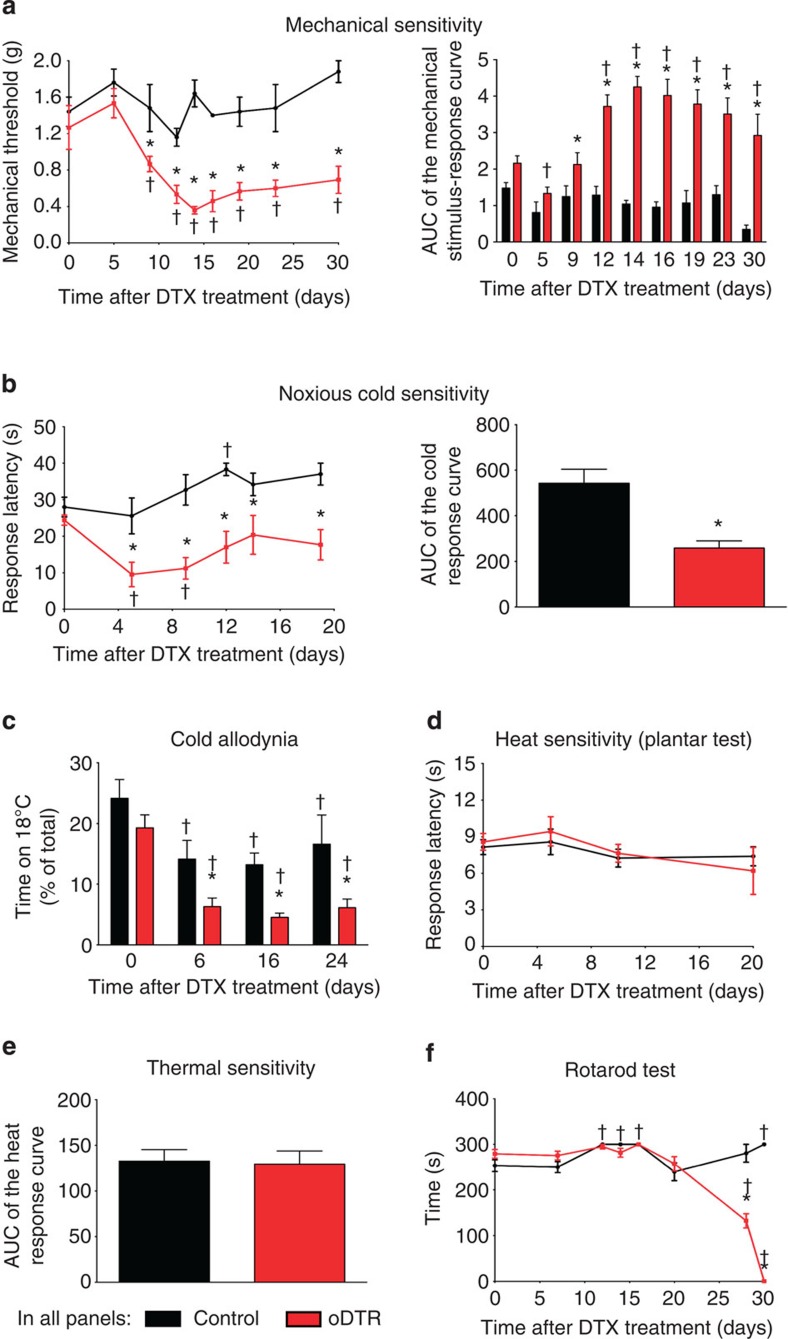
Overview of sensory dysfunction induced by oligodendrocyte ablation in genetically targeted mice following diphtheria toxin (DTX) treatment. (**a**) Threshold to mechanical von Frey stimuli applied to the paw plantar surface before and at various time points after DTX administration in mice with inducible oligodendrocyte ablation (oDTR) and DTX-treated control littermates. The area under the curve (AUC) of the curves representing frequency of responses to the intensity of applied von Frey stimuli is shown in the right hand panel to demonstrate sensitivity over the entire range of applied mechanical forces before and up to 30 days after DTX treatment (^†^*P*<0.05 as compared with the corresponding basal states; *P<0.05 as compared with control mice; repeated measures analysis of variance (ANOVA), *post-hoc* Fisher’s test; *n*=5 or 6 mice per group.). (**b**) Analysis of latency of withdrawal responses on a noxious cold plate (0 °C) in oDTR and control mice before and after DTX treatment. The AUC of each of the two curves shown in the left panel is represented in the right panel (*P<0.05, unpaired, two tailed, *t* test; *n*=7 mice per group.). (**c**) Analysis of sensitivity to non-noxious cold (18 °C) in terms of time spent on a 18 °C cold plate versus a plate at 30 °C. DTX-induced drop indicates development of hypersensitivity to non-noxious cold (cold allodynia; *n*=6 mice per group). (**d**,**e**) Analysis of latency of withdrawal responses to infrared heat applied to the plantar paw surface in oDTR and control mice. AUC for each of the individual curves shown in **d** is represented in **e** (*P<0.05, unpaired, two-tailed, *t* test; *n*=10 mice per group). (**f**) Analysis of motor function using the Rotarod test in oDTR and control mice before and up to 30 days after DTX treatment (*n*=5 mice per group). In all panels, unless otherwise indicated above, ^†^*P*<0.05 as compared with the corresponding basal states; *P<0.05 as compared with control mice; ANOVA, *post-hoc* Fisher’s test.

We then conducted a detailed characterization of the behavioural phenotype with respect to sensitivity to different somatosensory modalities, such as cold, mechanical touch, pressure and heat as well as analyses of motor function over 4–5 weeks after DTX injection. Importantly, key stages in nociceptive phenotypes were temporally matched with a thorough characterization of the pathophysiological sequelae of changes that occurred with respect to neuronal and glial function as well as axonal and myelin structure. Changes observed with respect to pathophysiology as well as behavioural phenotypes are summarized in Fig. 1b and described in detail in the following sections. Within a few days after administration of DTX, oDTR mice showed a striking deviation from normosensitivity to mechanical and cold stimuli, but not to heat (Fig. 2). Within 3 days after administration of DTX, oDTR mice, but not control mice, showed markedly reduced thresholds of withdrawal responses to mechanical punctuate (von Frey) stimuli applied to the plantar surface of hind paws. In these experiments, graded von Frey hairs (0.07–2 g) were applied to the plantar surface and withdrawal threshold was determined as the von Frey filament at which the animal withdrew its paw at least two times out of five applications (Fig. 2a; in all figures: *P<0.05 as compared with control; ^†^*P*<0.05 as compared with basal). Mechanical hypersensitivity was evident as a drop in the mechanical threshold starting from 6 days after DTX injection and reaching maximal values at 12–14 days after DTX injection (Fig. 2a, left hand panel). To comprehensively present all data on response frequencies to graded von Frey hairs over all forces tested, we constructed a response frequency versus stimulus intensity (that is, von Frey force applied) curve per group for every time point tested and calculated the area under the curve (AUC). Whereas the AUC remained constant in control mice over 30 days following DTX injection, oDTR mice demonstrated a significant increase in the AUC starting from 9 days after DTX injection (Fig. 2a, right hand panel).

Within 5 days after DTX administration, oDTR mice, but not control mice, showed markedly reduced response latencies to noxious cold stimulation applied to the hind paw plantar surface using a cold plate maintained at 0 °C (time course shown in Fig. 2b, left hand panel and integral of all latencies over 20 days is shown in the right hand panel of Fig. 2b). Thus, oDTR mice demonstrated significant hypersensitivity to noxious cold. To judge whether these phenotypic differences extended to non-noxious cold stimuli, we performed a thermal place preference test allowing mice to freely choose spending time on a plate at 30 °C versus a plate maintained at 18 °C. Before administration of DTX, oDTR and control mice showed a preference for 30 °C over 18 °C and were not significantly different from each other (Fig. 2c). Starting from 6 days after DTX injection, oDTR mice, but not control mice, showed a further, marked decrease in the time spent at 18 °C, indicating the development of significant cold allodynia (Fig. 2c).

Interestingly, this hypersensitivity to cold was not mirrored in responsiveness to heat stimuli. Upon using the plantar test apparatus to test response latency to infrared heat applied to the plantar surface of the hind paw, we observed no differences between oDTR mice and control mice before and after DTX administration over the entire course (time course is shown in Fig. 2d and AUC in Fig. 2e; *P*>0.05). The differential development of hypersensitivity to cold and heat stimuli indicates that the observed behavioural phenotype is not merely a result of hyperreflexia, but truly represents a sensory dysfunction. In order to test motor coordination following DTX delivery, we employed the rotarod test in oDTR mice and control mice and observed that starting from 30 days after DTX injection, oDTR mice demonstrated a sharp decline in the time spent on the rotating rod, indicating a major deficit in motor coordination (Fig. 2f).

In summary, oDTR mice, but not control mice, demonstrated a specific behavioral phenotype consisting of cold allodynia starting at 5 days post DTX, mechanical hypersensitivity which was marked at 12 days post DTX and a sharp decline in motor function at later stages after DTX injection (beginning at about 28 days).

### Time course of oligodendrocyte loss in the spinal cord

**Figure 3:**
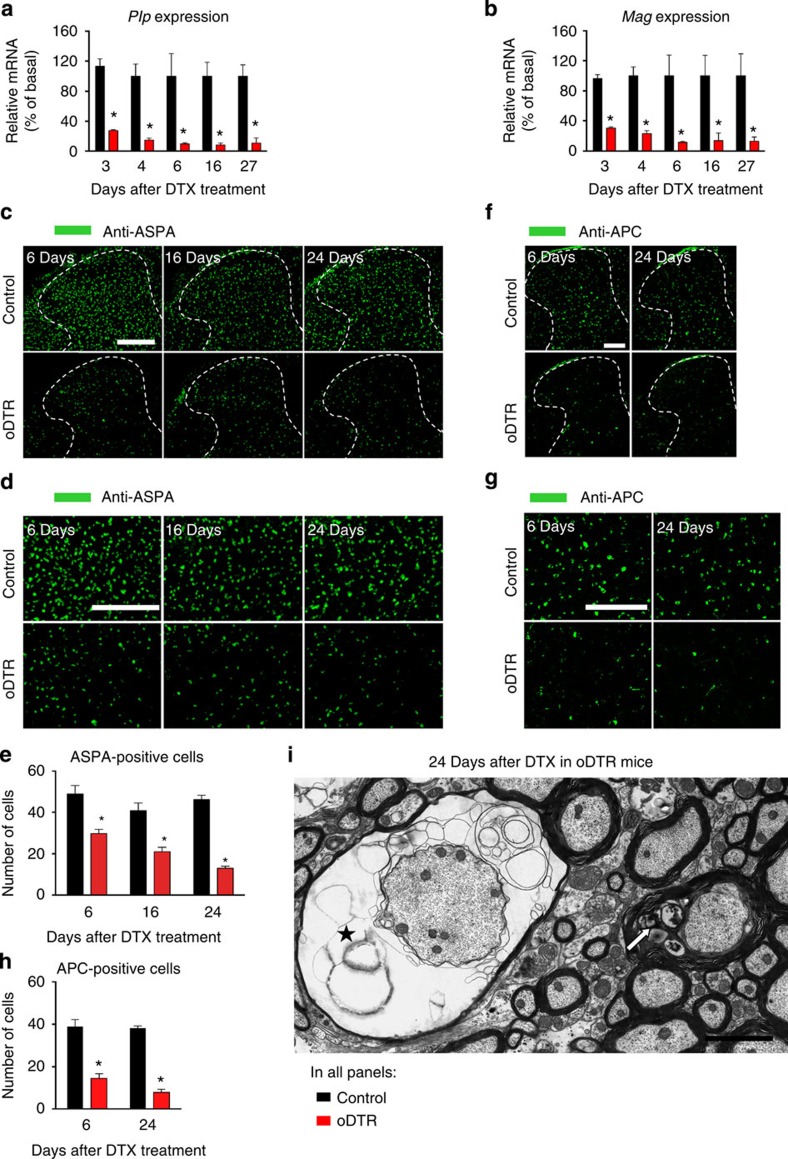
Analysis of spinal expression of oligodendrocyte markers and oligodendrocyte morphology in mice with inducible oligodendrocyte ablation (oDTR) and control littermates after diphtheria toxin (DTX) treatment. (**a**,**b**) Reverse transcription quantitative PCR analyses (qRT–PCR) of typical oligodendrocyte-enriched mRNAs in spinal cords of oDTR and control mice before and at 3–27 days post DTX treatment (*P<0.05 between the two groups at the indicated points; analysis of variance (ANOVA) of random measures, *post-hoc* Fisher’s test; *n*=2–5 mice per group). (**c**–**e**) Immunohistochemistry with an antibody recognizing ASPA, a protein expressed in somata of oligodendrocytes, on spinal cords of DTX-treated oDTR mice in comparison to DTX-treated control mice; spinal dorsal horn and surrounding white matter is shown in **c** and images in **d** represent high-magnification images from the white matter region. The corresponding quantitative analysis of ASPA-positive cells is shown in **e** (*P<0.05 between the two groups at the indicated points; ANOVA of random measures, *post-hoc* Fisher’s test; *n*=3 mice per group). (**f**–**h**) Immunohistochemistry with an antibody recognizing APC (CC1 clone), a protein expressed in somata of oligodendrocytes, on spinal cords of DTX-treated oDTR mice in comparison to DTX-treated control mice; spinal dorsal horn and surrounding white matter is shown in **f** and images in **g** represent high-magnification images from the white matter region. The corresponding quantitative analysis of APC-positive cells is shown in **h** (*P<0.05 between the two groups at the indicated points; ANOVA of random measures, *post-hoc* Fisher’s test; *n*=3 mice per group). (**i**) Electron microscopy (EM) analyses revealed diffuse presence of demyelinating axons in the spinal cord over late stages post-DTX in oDTR mice; shown are examples of an axon with myelin loss (asterisk) and an axon with a swollen inner tongue (white arrow) at 24 days post DTX. Scale bar, 300 μm in **c**, 150 μm in **d**,**f** and **g** and 2 μm in **i**.

To explore temporal links between the striking behavioural changes observed in DTX-treated oDTR mice and the ensuing pathology of oligodendrocytes in the spinal cord, we undertook several approaches. Reverse transcription quantitative PCR analyses (qRT–PCR) on spinal cords derived from oDTR mice and control mice treated with DTX revealed that the abundance of classical oligodendrocyte-enriched mRNAs, such as those encoding proteolipid protein and myelin-associated glycoprotein, decreased markedly and rapidly after administration of DTX in oDTR mice—a significant decrease was evident already when tested at 3 days post DTX in oDTR mice as compared with control mice (Fig. 3a,b). These results indicate that oligodendrocyte dysfunction in the spinal cord is initiated rather quickly after DTX administration in oDTR mice, that is, before the start of the sensory dysfunction. To trace the temporal profile of oligodendrocyte death, we performed immunohistochemical staining for ASPA, an antigen which is specifically expressed in oligodendrocyte somata (typical images from spinal dorsal horn shown in Fig. 3c, which are magnified in Fig. 3d, and quantitative summary shown in Fig. 3e). Whereas control mice maintained equivalent numbers of ASPA-positive cells in the spinal cord over the entire time course tested, oDTR mice displayed a significant decrease in ASPA-positive cells when tested first at 6 days post DTX and this loss increased progressively until over analysis up to 24 days after DTX administration (Fig. 3c–e). To further consolidate these analyses, we performed immunohistochemical staining for another antigen specifically expressed in oligodendrocyte somata, namely adenomatous polyposis coli (APC), using the prevalidated CC1 clone^11^), which revealed an identical pattern of changes as ASPA (Fig. 3f–h).

Loss of oligodendrocytes using the DTR-DTX system has been demonstrated previously, with a majority of analyses being focused on the brain^7^,^8^,^9^,^10^,^11^. Taken together with these studies, our observations from RT–PCR analyses and immunohistochemistry on a variety of independent markers of oligodendrocytes thus confirmed an oligodendrocyte loss in the spinal cord of DTX-treated oDTR mice. To further confirm a loss of oligodendrocytes, we performed electron microscopy (EM) analyses on spinal cords of DTX-treated control and oDTR mice. At 24 days post DTX, build-up of vacuolization and loss of myelin was evident in a diffuse manner over the spinal white matter in oDTR mice (examples of axons undergoing meylin pathology and demyelination are shown in Fig. 3i), which progressively intensified with time post DTX.

### Time course of myelin pathology in the spinal cord

**Figure 4:**
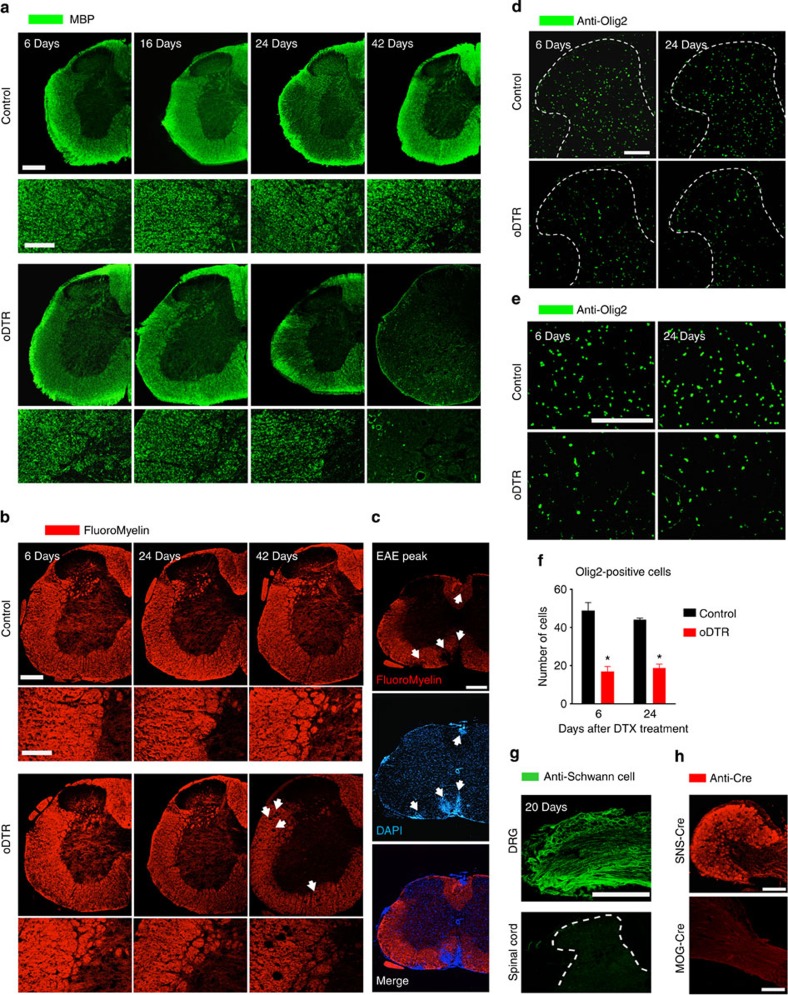
Analysis of changes in myelination in the spinal cord upon oligodendrocyte ablation (oDTR) after administration of diphtheria toxin (DTX) and potential modulatory factors. (**a**) The immunoreactivity for a myelin marker protein, MBP, remained unaltered in DTX-treated oDTR mice as compared with control mice until 24 days post DTX, but dropped thereafter (42 day time point shown). (**b**,**c**) FluoroMyelin staining in spinal cord failed to reveal demyelination in DTX-treated oDTR mice until 24 days post DTX, but was decreased at 42 days post DTX; decrease in FluroMyelin staining in plaques corresponding to immune cell infiltrates in spinal cords of mice with experimental autoimmune encephalomyelitis (EAE) is shown as proof-of-principle validation of FluoroMyelin staining. (**d**–**f**) Decrease in the immunoreactivity for Olig2, a marker of mature oligodendrocytes as well as oligodendrocyte precursors, in oDTR mice as compared with control mice. Spinal dorsal horn and surrounding white matter are shown in **d** and images in **e** represent high-magnification images from the white matter region. The corresponding quantitative analysis of Olig2-positive cells is shown in **f** (*P<0.05 between the two groups at the indicated points; analysis of variance of random measures, *post-hoc* Fisher’s test; *n*=3 mice per group). (**g**) Immunohistochemistry with an antibody directed against human peripheral nerve extract (anti-Schwann cell epitope) yields staining in dorsal root ganglia (DRG), but not in the spinal cords of DTX-treated oDTR mice. (**h**) Lack of expression of Cre recombinase in peripheral sensory neurons in Mog-Cre mice upon immunofluorescence analysis performed on the DRG; expression of Cre recombinase in the DRG of the SNS-Cre line is shown as a positive control for validation of the anti-Cre antibody. Scale bars represent 300 μm in **a**,**b**,**c** (low magnification) and **g**, 150 μm in **a**,**b** (high magnification) **d**,**e** and **h** (lower), and 50 μm in **h** (upper).

As expected, ablation of oligodendrocytes leads to a disruption of myelin. However, previous studies have reported that a loss of myelin in major tracts of the brain becomes evident starting only several days–weeks after ablation of oligodendrocytes in oDTR mice^7^,^8^,^9^,^10^,^11^. Indeed, we observed a dramatic decline in the immunoreactivity for typical myelin proteins, such as MBP, in the spinal cord of oDTR mice at 42 days post-DTX treatment in comparison to DTX-treated control mice (typical examples in Fig. 4a); this, however, had a late onset and indeed, anti-MBP immunoreactivity was comparable across control and DTR mice up to 24 days after DTX administration. Furthermore, FluoroMyelin staining was intact in oDTR mice when tested up to 24 days post DTX, but showed disruption of myelin at 42 days in oDTR mice post DTX (Fig. 4b). As a positive control for the validity of myelin staining using the above technique, we utilized spinal cords of mice immunized with the oligodendrocyte antigen, MOG, in the experimental autoimmune encephalomyelitis (EAE) model (Supplementary Fig. 1) and observed a clear disruption of myelin staining corresponding precisely to the loci of immune infiltrates in the spinal cord (Fig. 4c), which are characteristic to EAE. These results indicated that over time periods over which the nociceptive behavioural phenotype became apparent (that is, 6–12 days post-DTX), myelin disruption was not obviously visible in oDTR mice, but came about at later time points, as would be expected from oligodendrocyte loss.

Preservation of myelin despite loss of mature oligodendrocytes post-DTX over the early time points corresponding to nociceptive hypersensitivity could potentially result from recruitment of proliferating and newly differentiating oligodendrocytes. Therefore, we performed spinal immunohistochemistry for Olig2, a well-characterized marker protein for oligodendrocyte precursors as well as mature oligodendrocytes^10^ (typical examples in Fig. 4d, magnified image in Fig. 4e and quantification in Fig. 4f). Post DTX, we observed a net decrease in Olig2-expressing cells in oDTR mice as compared with control mice, which was very similar in time course and magnitude to the other oligodendrocyte marker proteins analysed earlier (Fig. 3). A lack of proliferating oligodendrocytes was also confirmed in subsequent analyses using markers of cell proliferation (below).

It has been reported that a loss of myelin in the CNS may be compensated by a migration of Schwann cells from the periphery into the CNS^12^. However, we observed that this scenario did not contribute to the maintenance of myelin after DTX administration because immunoreactivity for the Schwann cell marker Schwann/2E-antigen^12^ (DRG shown as positive control in Fig. 4g upper panel) was not evident in the spinal cord of oDTR mice treated with DTX (Fig. 4g, lower panel). Finally, we also confirmed that the MOG-Cre line, which we used to ablate oligodendrocytes, does not recombine in the peripheral nervous system by performing immunohistochemistry with an anti-Cre antibody on the DRG with attached nerve roots (Fig. 4h lower panel) compared with SNS-Cre mice^13^, which serve as a positive control for anti-Cre immunostaining (Fig. 4h upper panel).

Taken together, these analyses indicate that the early post-DTX time periods, which are associated with the pain phenotype in oDTR mice, are characterized by pathology and loss of oligodendrocyte somata, a relative preservation of myelin and lack of compensation from oligodendrocyte precursors or invading Schwann cells. At late time points post DTX (tested here at 42 days), oDTR mice show a clear disruption of myelin in light microscopic analysis as well as EM, which is accompanied by sensory as well as motor deficits in behavioural assays (above).

### Delayed innate immunity response in the spinal cord

**Figure 5:**
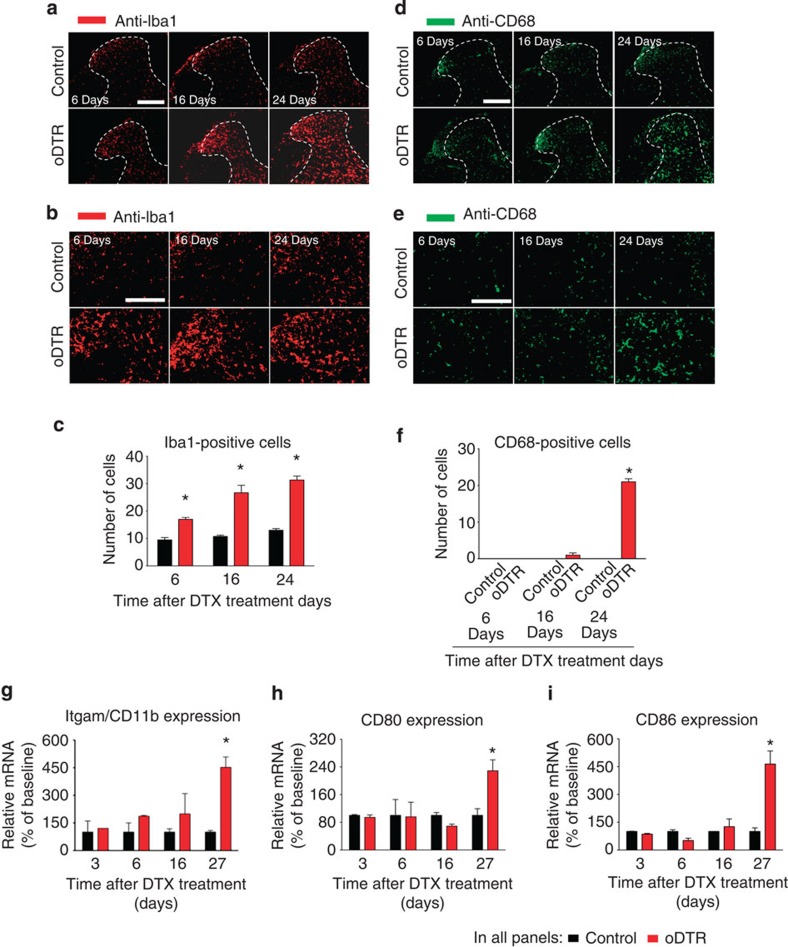
Analysis of microglial and astrocytic changes in mice upon oligodendrocyte ablation (oDTR) after administration of diphtheria toxin (DTX). (**a**–**c**) Immunohistochemistry with an antibody recognizing a microglial protein (Iba1) demonstrates an increase in microglial density at 16 and 24 days after DTX administration in oDTR mice but not in treated control mice. Images in **b** represent high-magnification view of the spinal dorsal horn. The corresponding quantification of Iba1-positive cells in the spinal dorsal horn over an area of 22,500 μm^2^ is represented in **c**. (**d**–**f**) Immunohistochemical analysis of CD68, a protein labelling phagocytic microglia, demonstrating microglial transformation at 24 days after DTX administration in oDTR mice but not in corresponding control mice. Images in **e** represent high-magnificantion view of the the spinal dorsal horn. The quantification of CD68-positive cells over an area of 22,500 μm^2^ in the spinal dorsal horn is shown in **f**. (**g**–**i**) Quantitative RT–PCR analysis of the expression of mRNAs for various microglial markers, indicating transformation of microglia in DTX-treated oDTR mice. Data were analysed via analysis of variance of random measures followed by *post-hoc* Fisher’s test; *n*=3 or 4 mice per group; *P<0.05. Scale bars represent 300 μm in **a** and **d** and 150 μm in **b** and **e**.

We then addressed whether an immune response caused by oligodendrocyte death could contribute to nociceptive hypersensitivity observed over 3–6 days onwards post-DTX in oDTR mice. Immunostaining of microglia in the spinal cord with Iba-1 indicated a significant increase in microglial numbers in oDTR mice over control mice starting at day 6 after DTX injection (typical examples are shown in Fig. 5a, magnified view of the dorsal horn in Fig. 5b and quantitative summary in Fig. 5c). To address whether microglial activation was associated with microglial transformation, we performed immunohistochemistry with anti-CD68 antibody, which identifies phagocytic microglia^14^. These experiments revealed that CD68 immunoreactivity increases significantly starting only at day 24 after DTX injection in oDTR mice over DTX-injected control mice, suggesting delayed microglial transformation (typical examples shown in Fig. 5d, magnified view of the dorsal horn in Fig. 5e and quantitative summary in Fig. 5f).

These results were further confirmed by qRT–PCR analyses on spinal cord of DTX-treated oDTR and control mice. Itgam/CD11b, a marker for microglia^15^, was observed to be increased significantly in expression in oDTR mice as compared with control mice at day 27, but not at prior time points, post-DTX (Fig. 5g). Furthermore, mRNAs for co-stimulatory proinflammatory molecules expressed in microglia, namely CD80 and CD86 (ref. 15), were observed to be elevated in oDTR mice as compared with control mice at, but not before, day 27 post DTX (Fig. 5h,i). It should be noted that none of the above markers distinguish between microglia and monocytes^15^.

**Figure 6:**
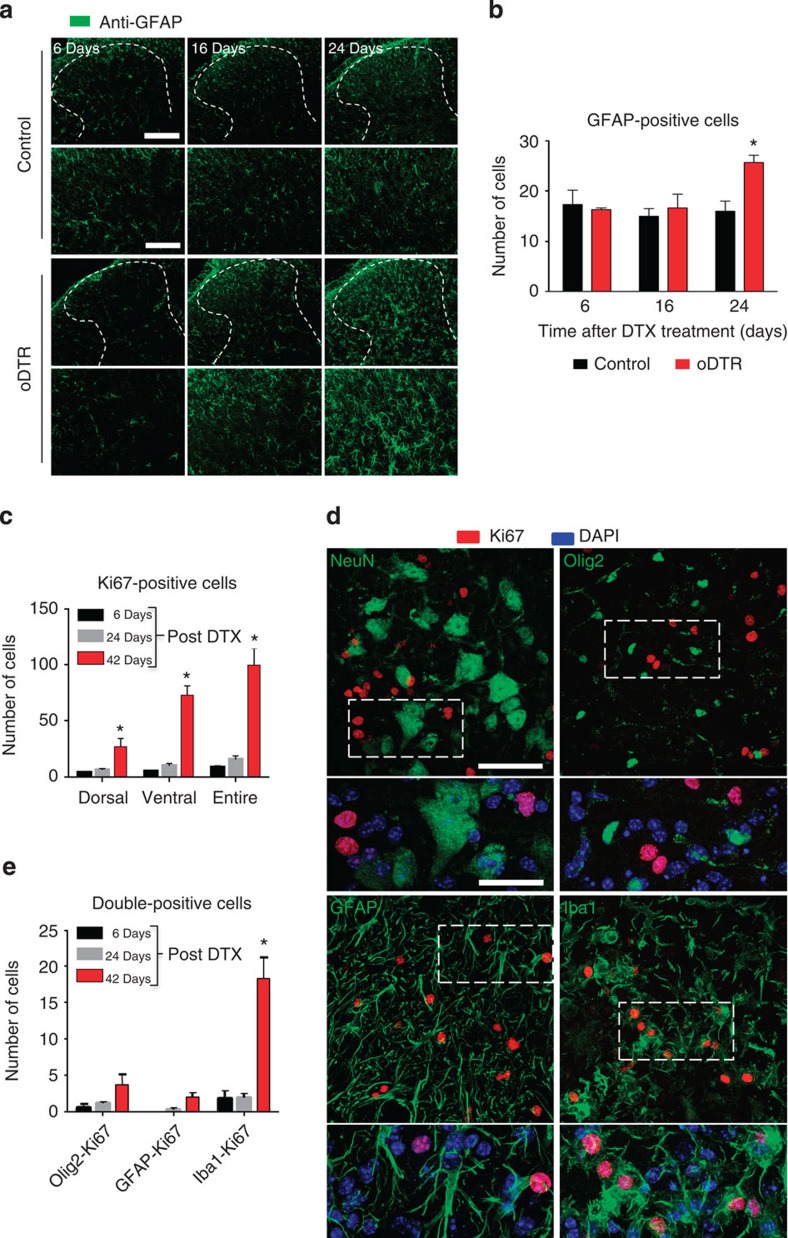
Analysis of astrogliosis and cell proliferation in spinal cords of mice with diphtheria toxin (DTX)-induced oligodendrocyte ablation (oDTR). (**a**,**b**) Immunohistochemical analysis of a marker for astroglia (GFAP) indicates astrogliosis at 24 days after DTX administration in oDTR mice but not corresponding control mice. Lowermost panels show a higher-magnification view of activated astroglia. The corresponding quantification of GFAP-positive cells over an area of 22,500 μm^2^ is shown in **b**. (**c**) Analysis of immunoreactivity for Ki-67, a marker for proliferating cells in oDTR mice at various time points post DTX quantified over regions of interest of 22,500 μm^2^ in the spinal dorsal horn and white matter. (**d**) Double-immunofluorecence confocal analysis on spinal cords of DTX-treated oDTR mice to determine the nature of proliferating cells, using markers for neurons (NeuN), oligodendrocytes (Olig2), microglia (Iba1) and astrocytes (GFAP), with Ki-67. Magnified images of area within dashed square are shown right underneath; please note co-immunoreactivity of Ki67 with Iba1, which was further confirmed by nuclear co-localization of Ki67 with the DAPI-positive nucleus of the microglial cell. (**e**) Quantification of double-positive cells expressing various markers and Ki67, corresponding to examples shown in **d**, quantified over regions of interest of 22,500 μm^2^ in the spinal dorsal horn and white matter. Data were analysed via analysis of variance of random measures followed by *post-hoc* Fisher’s test; *n*=3 or 4 mice per group; *P<0.05. Scale bars represent 300 μm in **a** and 150 μm in lowermost panels of **a**. Scale bars represent 50 μm in **d** (low magnification) and 25 μm in **d** (high magnification).

Similarly, immunostaining with an anti-GFAP antibody revealed that the number of GFAP-positive astrocytes increases significantly only at late stages (24 days after DTX treatment) in oDTR mice as compared with DTX-treated control mice (typical examples in Fig. 6a (upper row), magnified view of the dorsal horn in Fig. 6a (lower row) and quantitative summary in Fig. 6b). At 24 days after DTX injection in oDTR mice, but not at prior time points, we observed morphological changes in astrocytes indicative or reactive astrogliosis (Fig. 6b).

To directly address whether the above described changes in microglia and astrocytes truly reflect increased proliferation, we performed dual immunofluorescence for Ki67, a marker of proliferating cells (Fig. 6c–e). A significant rise in the number of proliferating cells was revealed by anti-Ki-67 immunoreactivity in oDTR mice, both in the dorsal as well as the ventral horns, at 42 days post DTX as compared with 6 or 24 days post-DTX. Co-immunostaining revealed a lack of co-localization of nuclei of neurons (identified via NeuN staining) and Ki67 staining (examples in Fig. 6d). Similarly, neither Olig2-positive nuclei of oligodendrocytes and oligodendrocyte precursors nor the nuclei of GFAP-positive astrocytes co-localized with Ki67 immunoreactivity (examples in Fig. 6d). Instead, co-localization was observed between the nuclei of microglia (identified via Iba1 staining) and Ki67 immunoreactivity (examples in Fig. 6d). Thus, among the above cell populations studied in the spinal cord of DTX-treated oDTR mice, only microglia demonstrated a significant proliferation starting at day 42 post DTX, whereas astrocytes showed an increase in GFAP staining intensity and shape change, but no obvious proliferation (quantitative summary in Fig. 6e). Importantly, oligodendrocyte precursors were not observed to show proliferation at this time point.

Thus, oligodendrocyte ablation is associated with microglial proliferation and astroglial morphological changes starting at time points, which are considerably delayed in comparison to the development of the mechanical nociceptive hypersensitivity and cold allodynia/hyperalgesia.

### Lack of adaptive immunity after oligodendrocyte ablation

**Figure 7:**
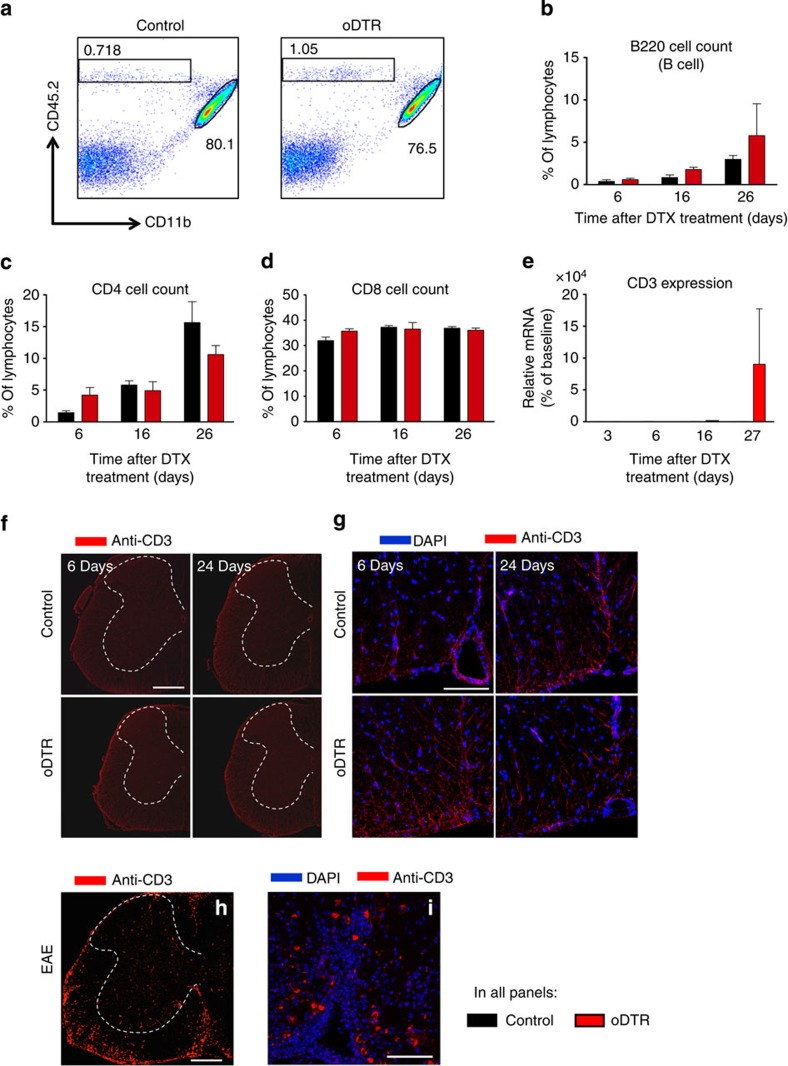
Analysis of lymphocyte infiltration in the CNS of mice with inducible oligodendrocyte ablation (oDTR) and corresponding control littermates after administration of diphtheria toxin (DTX). (**a**) Representative plots of cells isolated from the spinal cord of DTX-treated mice 6 days post DTX injection. Percentages of invading cells (CD45hi, CD11b-) and microglia (CD45int, CD11bhi) are shown. (**b**) Flow cytometric analysis of cells isolated from the spinal cord of DTX-treated mice indicates no change in percentage of B-cell infiltration in oDTR mice as compared with control mice. (**c**,**d**) Flow cytometric analysis demonstrating no change in percentage of T cells in the spinal cord of oDTR mice as compared with corresponding control mice after DTX administration. (**e**) Quantitative RT–PCR expression analysis on CD3 mRNA performed on lumbar spinal cords of DTX-treated control mice and oDTR mice. (**f**,**g**) Immunofluorescence analyses showing lack of difference in the abundance of CD3-expressing T cells in spinal cords of DTX-treated control mice and oDTR mice. Images in **g** represent high-magnificantion view of the white matter. (**h**,**i**) Infiltration of CD3-expressing T cells in spinal cords of mice with experimental autoimmune encephalomyelitis (EAE) is shown as a positive control for validation of the anti-CD3 staining. Images in **i** represent high-magnification view of the the white matter. In **b**–**e**, data were analysed via analysis of variance followed by *post-hoc* Fisher’s test; *n*=3 or 4 mice per group; *P<0.05. Scale bars represent 300 μm in **f** and **h**, and 100 μm in **g** and **i**.

To address whether an adaptive immune response is evoked in the spinal cord by DTX-induced oligodendrocyte cell death, we performed a flow cytometric analysis of cells isolated from the spinal cord of oDTR and control mice at various time points after DTX administration. Figure 7a shows an example of an experiment sorting percentages of lymphocytes, which are classified here as CD45-high, CD11b-negative (indicated by square box) and microglia, classified here as CD45-intermediate and CD11b-high (indicated by elliptical shape) from spinal cord of DTX-treated oDTR (panel at right) or control mice (panel at left) at day 6 post-DTX; no significant differences in these cell populations are observed between the two groups. Lymphocytes were further broken down in B cells (B220, Fig. 7b) and T cells (expressing CD4 or CD8, Fig. 7c,d); these populations were not significantly different between the two groups when tested at 6, 16 or 24 days post DTX (Fig. 7b–d).

To further confirm these results, we performed RT–PCR analysis on the expression of CD-3, a T-lymphocyte marker, which was not significantly different between control and oDTR mice up to 27 days post DTX (Fig. 7e). Furthermore, we also performed immunostaining using an anti-CD3 antibody, which broadly detects T-lymphocytes. Spinal cord white matter from DTX-treated oDTR mice did not show any increase in anti-CD3 immunoreactivity at any stage post DTX as compared with DTX-treated control mice (typical examples in Fig. 7f and higher magnification in Fig. 7g); whereas in the EAE spinal cord, which served as a positive control, a significant amount of CD3-positive immunoreactivity was observed particularly in the white matter (typical example shown in Fig. 7h; higher magnification of white matter in Fig. 7i; also see supplementary Fig. 1). These diverse approaches indicate that oligodendrocyte ablation in oDTR animals is not associated with anti-CNS immunity in the spinal cord, at least over the time points analysed that were associated with the development of central neuropathic pain.

### No involvement of microgliosis and T-lymphocyte infiltration

**Figure 8:**
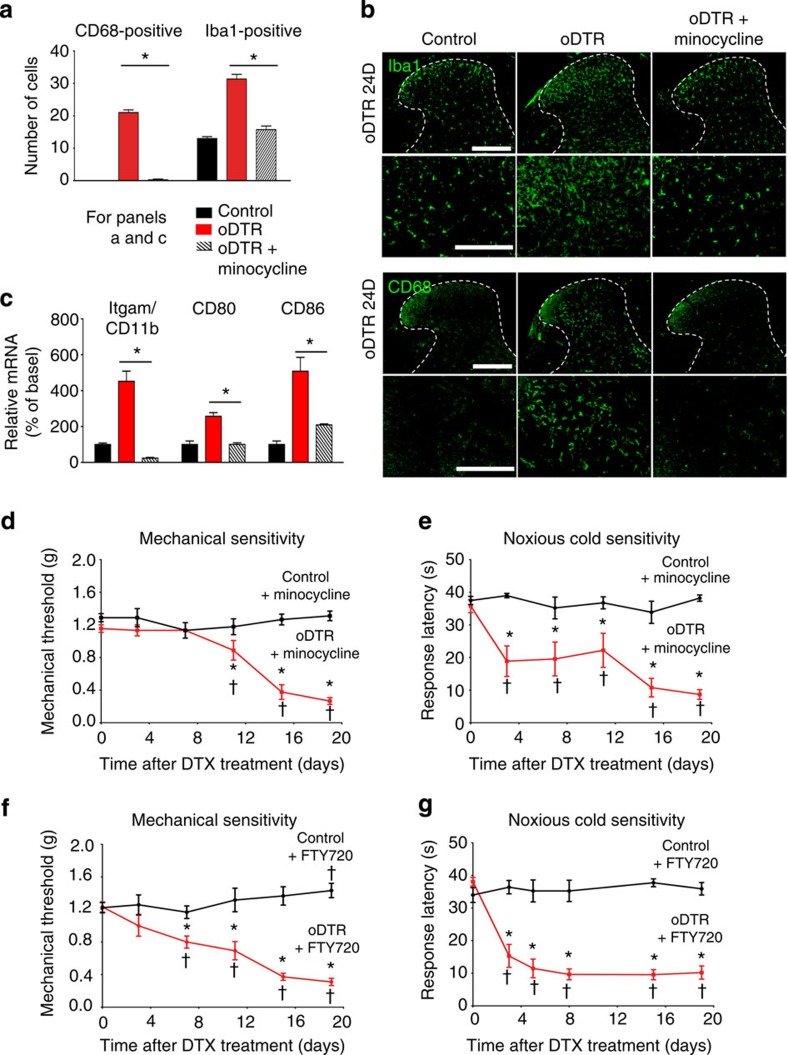
Blocking microglial transformation or T-lymphocyte infiltration in the spinal cord does not inhibit pain hypersensitivity induced by oligodendrocyte ablation in oDTR mice. (**a**,**b**) Demonstration of the efficacy of minocycline in inhibiting microglial activation and transformation in the spinal cord of DTX-treated oDTR mice via immunohistochemistry with antibodies recognizing CD68 and Iba1. Quantification over regions of interest of 22,500 μm^2^ in the spinal dorsal horn and white matter is shown in **a** and typical examples are shown in **b**. (**c**) Further evidence for efficacy of minocycline in inhibiting microglial transformation via quantitative RT–PCR analysis on markers of microglial activation. In **a** and **c**, *P<0.05 between the two groups at the indicated points; analysis of variance (ANOVA) of random measures followed by *post-hoc* Fisher’s test; *n*=3 or 4 mice per group. (**d**) DTX-induced drop in the mechanical thresholds (mechanical hypersensitivity) is preserved in minocycline-treated oDTR mice as compared with corresponding control mice. (**e**) Hypersensitivity to noxious cold stimuli (0 °C) is preserved in DTX-treated oDTR mice receiving minocycline as compared with corresponding control mice. (**f**,**g**) DTX-induced drop in mechanical thresholds (mechanical hypersensitivity, **f**) as well as drop in response latency to noxious cold (**g**) is preserved in DTX-treated oDTR mice receiving FTY720 as compared with the corresponding control mice. Data were analysed via ANOVA, *post-hoc* Fisher’s test in **d**–**g**; *n*=9–12 mice per group; ^†^*P*<0.05 as compared with the corresponding basal states; *P<0.05 as compared with control mice. Scale bars represent 300 μm in **b** (low magnification), and 150 μm in panels with high magnification.

Spinal microgliosis as well as T-cell infiltration have been linked to nociceptive hypersensitivity, which develops following peripheral nerve injury^16^. Therefore, we sought to functionally address whether minimal contributions of microglial or T-cell components, which may have escaped detection in oDTR mice, could account for the sensory phenotype. Minocycline, a drug that inhibits microglial function^17^, was given systemically twice a day over the entire course of DTX administration and continued 20 days after DTX injection. In DTX-treated oDTR mice, microglial proliferation (Iba1 immunoreactivity) as well as microglial transformation (CD68 immunoreactivity) was significantly and completely inhibited when minocycline was administered (see Fig. 8a for quantitative summary of stained microglia, typical examples of immunohistochemistry are shown in Fig. 8b). Moreover, qRT–PCR analyses confirmed a marked and significant reduction in the expression of markers of microglial activation in Minocycline-treated oDTR mice post DTX, such as Itgam/CD11b, CD80 and CD86 (Fig. 8c). However, neither the induction nor the maintenance of mechanical hypersensitivity nor cold allodynia/hyperalgesia induced by oligodendrocyte ablation in oDTR mice was significantly affected upon minocycline delivery (Fig. 8d,e). Similarly, administration of FTY720, an inhibitor of T-lymphocyte infiltration^18^, failed to influence nociceptive hypersensitivity in DTX-treated oDTR mice (Fig. 8f,g); in parallel experiments, we ascertained that FTY720 treatment abolished T-cell infiltration in the spinal cord of EAE mice, thereby providing evidence for the efficacy of the FTY720 regimen employed (Supplementary Fig. 1). Thus, neither microgliosis nor T-lymphocyte infiltration are causally associated with nociceptive hypersensitivity developing from oligodendrocyte ablation.

### Pathophysiological changes in neuronal somata and axons

**Figure 9:**
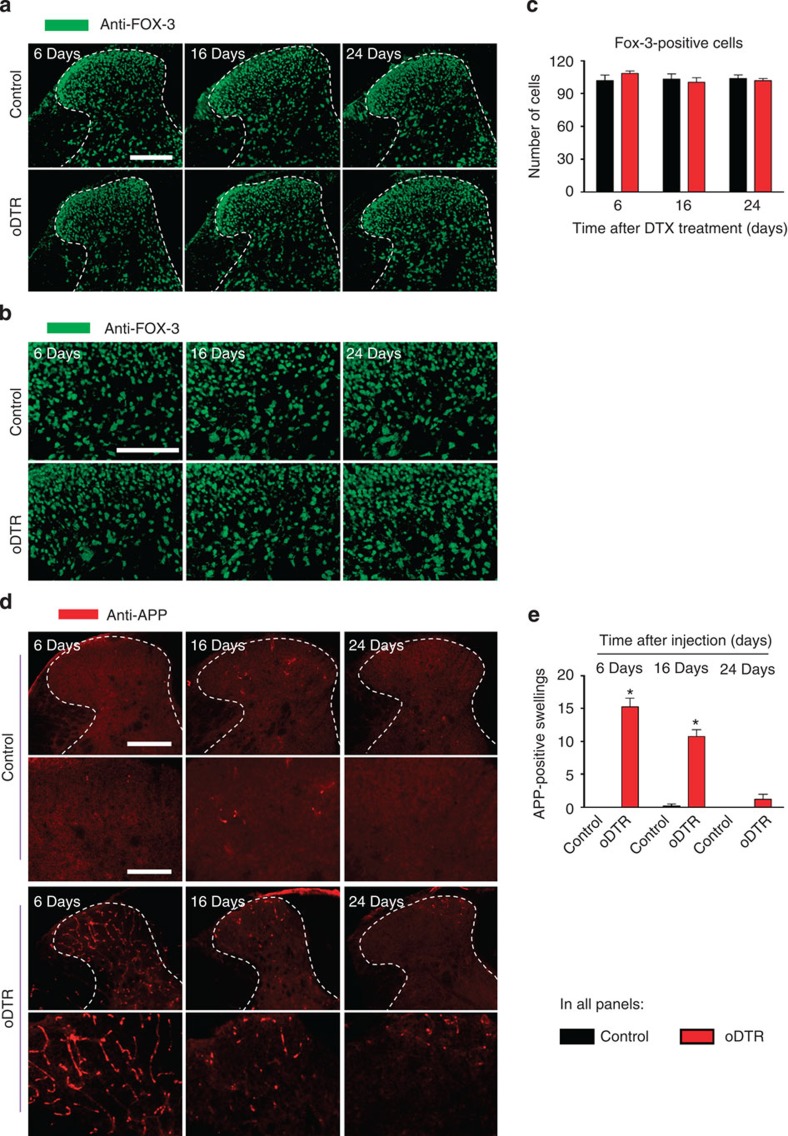
Analysis of neuron-specific markers in mice with inducible oligodendrocyte ablation (oDTR) as compared with control mice. (**a**–**c**) Immunohistochemistry with an antibody-recognizing FOX-3, a neuronal marker, indicates lack of change in neuronal density in DTX-treated oDTR mice as compared with corresponding control mice. spinal dorsal horn and surrounding white matter are shown in **a** and images in **b** represent high-magnification images from the grey matter region. The corresponding quantitative analysis of FOX-3-positive cells over an area of 22,500 μm^2^ is shown in **c**. (**d**,**e**) DTX-treated oDTR mice show an early increase in APP expression, a marker of acute axonal injury via immunohistochemistry; higher-magnification view shown in lower panels. Corresponding quantification of APP immunoreactive axonal swellings over an area of 22,500 μm^2^ is represented in **e**. Data were analysed via analysis of variance of random measures followed by *post-hoc* Fisher’s test; *n*=3 or 4 mice per group; *P<0.05. Scale bar, 300 μm in **a**, and **d** (upper row, low magnification), and 150 μm in **b**, and **d** (lower row, high magnification).

We then analysed how neurons involved in spinal processing of nociception are affected upon progressive dysfunction and death of oligodendrocytes. Immunohistochemical analysis of Fox-3, which is a marker of neuronal nuclei, revealed no changes in neuronal distribution when analysed up to 24 days after DTX injection in oDTR mice as compared with DTX-treated control mice (typical examples in Fig. 9a, magnified images of the spinal dorsal horn in Fig. 9b and quantitative summary of stained cells in Fig. 9c). We then analysed immunoreactivity for APP, a protein that has been demonstrated to be a marker for acute axonal pathology as well as a pathophysiological inhibition of axonal transport^10^,^19^. APP immunoreactivity demonstrated a striking increase in oDTR mice over the early stages of DTX delivery as compared with DTX-treated control mice, which then recovered back to basal levels by 24 days after DTX delivery (see Fig. 9d for typical examples with magnified images of the spinal dorsal horn shown in lower panels and Fig. 9e for quantitative summary). Taken together, these data indicate that although nociceptive hypersensitivity is not accompanied by a widespread loss of neurons, oligodendrocyte ablation in the CNS induces a pathophysiological response in axons early on at time periods concurrent with the onset of pain hypersensitivity.

### Ultrastructural changes in the spinothalamic tract

**Figure 10:**
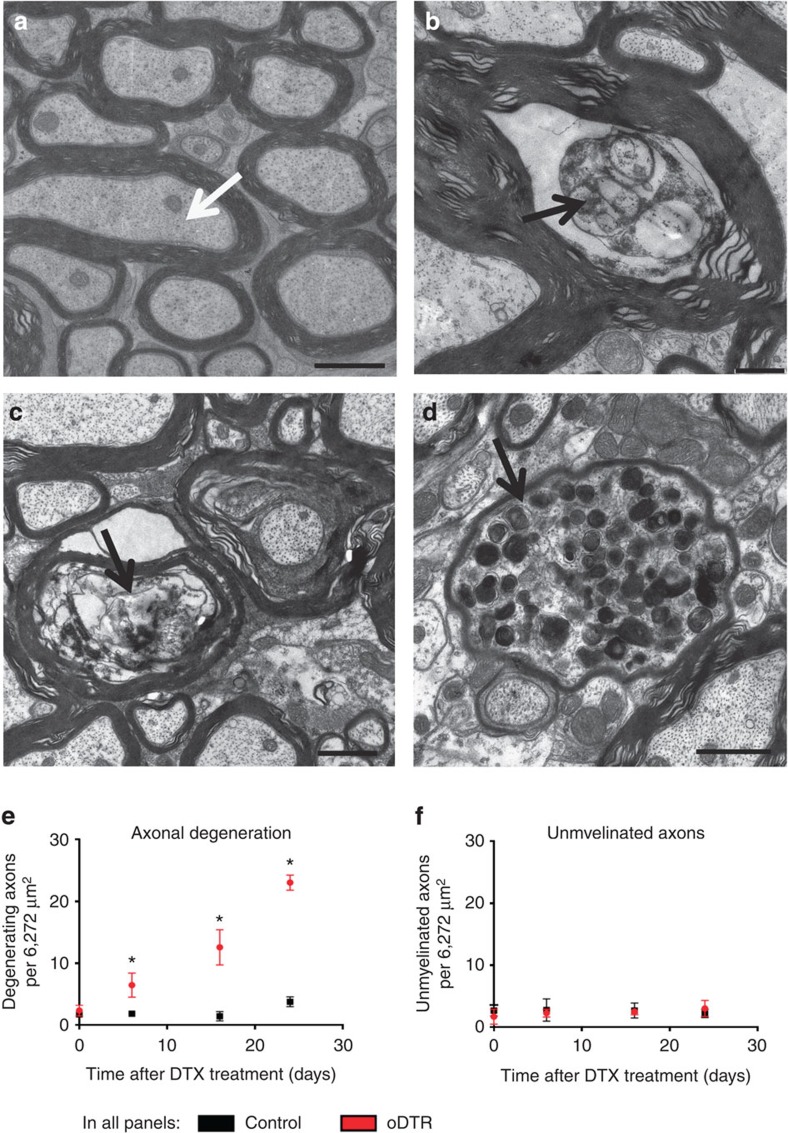
Quantitative and ultrastructural analysis of myelin integrity and axonal pathology in the spinalthalamic tract. (**a**) Example of ultrastructure of axons and myelin in the spinothalamic tract of DTX-treated control mice. (**b**–**d**) Ultrastructural analysis of the spinothalamic tract in DTX-treated oDTR mice showing progressive axonal pathology, evident as degenerating axons at 6 days (**b**) and 16 days (**c**) post DTX and axonal swelling (**d**). (**e**) Quantitative analysis of degenerating axons in the spinothalamic tract of DTX-treated oDTR mice corresponding to images shown in **b**–**d**. (**f**) Although examples of demyelination were diffusely observed from 16 days onwards in DTX-treated oDTR mice (Fig. 3i), quantitative analysis of unmyelinated axons in the spinothalamic tract showed no net significant change until 24 days in DTX-treated oDTR mice in comparison to DTX-treated control mice. Data were analysed via analysis of variance of random measures followed by *post-hoc* Fisher’s test; *n*=3–8 mice per group (**e**) and 3–5 mice per group (**f**); *P<0.05. Scale bars represent 1 μm in **a**,**c** and **d** and 500 nm in **b**.

Our observations on a transient increase of APP immunoreactivity in oDTR mice, indicative of acute axonal injury during the early stages of DTX delivery, prompted a detailed ultrastructural analysis of axonal integrity in the spinal nociceptive pathways. Strikingly, ultrastructural analysis of axons in the spinothalamic tract revealed a pronounced and progressive pattern of axonal degeneration and loss in oDTR mice, but not control mice, starting at early time points after DTX injection (a typical example from DTX-treated control mice are shown in Fig. 10a, examples from DTX-treated oDTR mice are shown in Fig. 10b–d, and quantitative summary of denegerating axons is shown in Fig. 10e). This was evident as degenerating axons in oDTR mice (Fig. 10b,c showing 6 days and 16 days post DTX, respectively) and axonal swelling (Fig. 10d), which corresponds to the APP pathology described above and shown in Fig. 9. Interestingly, these changes were already significant at the time of onset of nociceptive hypersensitivity and increased progressively in their frequency as nociceptive hypersensitivity increased with time (Fig. 10e).

As reported in Fig. 3, demyelination set in with a delay post-DTX and progressively, diffusely distributed axons with a swollen inner tongue of myelin became evident. However, the count of unmyelinated axons did not rise significantly up to 24 days after DTX administration in oDTR mice (Fig. 10f), consistent with reports on brain tracts, such as the corpus callosum^8^,^10^,^11^. Taken together, these results suggest that primary oligodendrocyte cell death results in rapid, early-onset axonal degeneration and myelin pathology and demyelination with a delayed time course.

## Discussion

Studies on genetically induced oligodendrocyte ablation^7^,^8^,^9^,^10^,^11^ have converged on a common set of phenotypic changes, despite small differences pertaining to onset and progression of the phenotype between individual models based upon genes targeted by the DTX-DTR system (oligodendrocyte specificity versus additional influence on Schwann cells) and differences in methodological aspects of the genetic ablation system (for exampl, constitutive versus inducible, adult-onset). The common phenotypic changes reported in the brain include a rapid loss of labelled oligodendrocyte somata, microglial reaction, astrogliosis, lack of adaptive immunity with absence of invading T cells, progressive and late demyelination and myelin pathology and neurological symptoms (ataxia, tremor, ultimately death). This observed sequale was also observed in the spinal cord in our study. However, the main finding of this study was that coincident with an early and widespread loss of oligodendrocytes, mice showed signs of exaggerated nociceptive sensitivity manifest as hyperalgesia (enhanced pain in response to noxious stimuli) and pain in response to non-noxious stimuli (allodynia). Importantly, this occurred at a time point that preceeded overt demyelination and ataxia and so on and coincided with early axonal pathology in the spinothalamic tract, including indications of axonal transport defects. Mechanistically, hyperalgesia and allodynia were not causally associated with microglial reaction or T-cell contributions.

Thus, this study indicates that a primary loss of oligodendrocytes independent of immune reactions can cause a nociceptive dysbalance. For decades, research on mechanisms of pain was primarily focused on neuronal mechanisms. Over the past few years, a large body of evidence has emerged pointing to important contributions for microglia and astrocytes to nociceptive modulation^1^,^2^,^4^. However, oligodendrocytes, which also comprise a highly prevalent cell type in the CNS, had not been studied in this regard so far. This study now demonstrates that oligodendrocyte function is required for normosensitivity to somatosensory stimuli. Here we report the observation that specific and targeted oligodendrocyte death in the adult CNS induces a highly specific pattern of sensory changes consisting of early and pronounced nociceptive hypersensitivity as well as a late and progressive decline in motor coordination. Interestingly, the pattern of nociceptive hypersensitivity comprised of hyperalgesia as well as allodynia to mechanical and cold stimulation, but not to heat, which is reflective of changes classically associated with neuropathic pain.

As expected, oligodendrocyte ablation leads to demyelination, which is evident as vacuolization and loss of myelin markers in histological analyses as well as neurological symptoms, such as ataxia and tremor in behavioural analyses. However, our systematic temporal analyses indicated a temporal mismatch between the onset of pain and overt demyelination after oligodendrocyte ablation. Thus, this throws some light on the long-standing question of whether demyelination is causally associated with pain. Demyelination constitutes a plausible hypothesis for pathological pain, as many scenarios can account for demyelinated axons becoming hyperexcitable, for exampl, because of an ionic dysbalance, despite a slowing of conduction velocities. However, it is noteworthy that in peripheral neuropathies, there is no clear correlation between demyelination and pain: demyelinating as well as non-demyelinating neuropathies can be accompanied by pain and not all demyelinating neuropathies are painful^20^. Our results on sensory abnormalities, taken together with previous studies, indicate that upon primary oligodendrocyte loss, progressive demyelination correlates temporally well with the onset of motor disturbances, but pain can precede overt demyelination.

Indeed, EM analyses as well as expression of marker proteins indicated that axonal pathology preceded demyelination and an innate immune response in the brain as well as in the spinal cord in this model and temporally matched the development of nociceptive hypersensitivity. Dysregulation of axonal transport, as indicated by an early accumulation of APP^10^,^19^ in axons of spinal dorsal horn neurons, was obvious in a precisely coordinated matter, which matched remarkably well to the progressive development of central neuropathic pain. Axonal degeneration was initiated concurrent with the development of nociceptive hypersensitivity and the extent of degenerating axons was significant, but small, over early stages. In contrast, defects in axonal transport were maximal at the time of induction of nociceptive hypersensitivity, which was also reflected in axonal swelling in EM analyses. It is intriguing how these changes could be related to oligodendrocyte loss. The emerging concept is that dynamic communication between oligodendrocytes and neurons is pivotal for neuronal function^5^. For example, it has been hypothesized that oligodendrocyte function and local glia-to-axon signalling may be required to ensure a persistent supply of ribosomes for local protein translation within ensheathed axons^5^. Thereby, a disturbance of local glial axon signalling may result in impairment of local axonal protein synthesis. These findings would tie in well with recent findings that local axonal translation is critically important in peripheral axons as well as in spinal neurons processing nociceptive stimuli^21^. Alternatively, a loss of oligodendrocytes may affect the metabolic status and upset energy balance in axons, as indicated in recent studies^22^.

Despite these insights, some aspects remain unclear. First, spontaneous pain was not analysed here; therefore, it remains unclear whether similar mechanisms apply across nociceptive hypersensitivity and spontaneous pain upon oligodendrocyte loss. Second, why and how axonal pathology in the spinal cord leads to changes in pain perception is unclear; it is conceivable that axonopathy in the spinothalamic tract disrupts the balance between excitation and inhibition in the thalamus, leading to hyperalgesia and allodynia. Spinothalamic tract lesions have been implicated in the development of different pain syndroms including central pain in syringomyelia^23^, spinal cord injury^24^ as well as central poststroke pain^25^. Furthermore, interruption of the STT has been shown to cause hyperactive bursting in the sensory thalamus in rats, primates and humans^26^,^27^,^28^.

The results described here have implications for pain in disorders involving oligodendrocyte loss and pathology, for example, multiple sclerosis (MS), spinal cord injury, among others. Interestingly, pain is a frequent component of the pathophysiological sequelae in MS and starts early on concurrent with the onset of other symptoms in MS^29^,^30^. The most common form of pain in MS is central neuropathic extremity pain^29^,^30^,^31^,^32^, which is distinguished by continuous burning pain^31^ as well as mechanical and cold allodynia^30^. Furthermore, nearly all MS patients who experience central pain report alterations in temperature or pain sensation in the affected region^29^, suggesting contributions of lesions in the spinothalamic tract. MS is characterized by the pathophysiological triad, namely that of oligodendrocyte death, CNS inflammation and demyelination, all culminating in axonal loss. Recent studies in the EAE model, including ours, have addressed and reported an involvement of immune mechanisms in MS-associated pain^33^,^34^. Here we found that primary oligodendrocyte loss in the absence of adaptive immunity can induce symptoms of central neuropathic pain, which are similar to those found in MS patients as described above. Interestingly, neither the microglia inhibitor Minocycline nor FTY720, an inhibitor of lymphocyte infiltration that is highly effective in the reducing CNS inflammation^18^, affected the development or maintenance of nociceptive hypersensitivity upon oligodendrocyte loss. These results suggest that targeting oligodendrocyte function and axonal pathology in addition to immune-based therapies may be of value in the context of pain treatment. This may also be relevant to pain in other central neuropathic disorders, for example, spinal cord injury^24^, which also involve injury-induced oligodendrocyte loss, defects in axonal transport and secondary axonal degeneration in addition to immune responses.

In summary, this study demonstrates that central neuropathic pain can be induced by oligodendrocyte death and axonal pathology in the absence of an innate or adaptive immune response. Our results suggest that it will be worthwhile to study in further detail defects in oligodendrocyte function and interactions between oligodendrocytes and neurons, particularly from the point of view of understanding and preventing defects in axonal transport, axonal pathology and central neuropathic pain.

## Methods

### Genetically modified mice

Homozygous mice harbouring the floxed allele of DTX receptor (*DTR*^*fl/fl*^) were crossed with *Mog-Cre*^*+ve*^ mice, which express the cre recombinase under the control of the oligodendrocyte-specific myelin oligodendrocyte glycoprotein (*mog*) gene promoter to obtain litters consisting of *DTR*^*fl/fl*^*;Mog-Cre*^*+ve*^ mice^6^ (referred to herein as oDTR mice) and *DTR*^*fl/fl*^ mice as well as *Mog-Cre*^*+ve*^ mice, which were used as control littermates (referred to herein as controls). Ablation of oligodendrocytes in adult oDTR mice was achieved by intraperitoneal injection of DTX on 3 consecutive days. All animal use procedures were in accordance with the ethical guidelines imposed by the local governing body (Regierungspräsidium Karlsruhe, Germany). Mice aged 4–51 weeks of both genders were used and matched for gender and age across groups.

### Administration of drugs

DTX (Sigma-Aldrich) was dissolved in 1 × PBS and injected intraperitoneally (25 ng per g body weight) on 3 consecutive days. FTY720 (Cayman Chemical) was dissolved at 20 mg ml^−1^ in dimethylsulphoxide. After dilution with distilled water, FTY720 (1 mg per kg body weight) was administered intravenously every 24 h for 20 days. Minocycline hydrochloride (Sigma-Aldrich) was dissolved in distilled water and injected intraperitoneally (30 mg per kg body weight) 16 and 0.5 h before the first DTX application. Afterwards, Minocycline was administrated two times daily for 20 days.

### Behavioural testing

Mice were habituated and three baseline measures were obtained for each behavioural test in separate sessions within the week before actual testing. The observer was fully blinded to the identity of the groups in all behavioural tests.

*Mechanical sensitivity*. Animals were placed on an elevated wire grid and mechanical sensitivity determined upon paw withdrawal to either application of graded force via a dynamic aesthesiometer (UgoBasile Inc.) or to manual application of graded von Frey hairs (0.07–2 g) to the plantar surface (see ref. 35 for details). Response frequency was calculated as the mean number of withdrawals out of five applications of the respective filament at 10 s intervals. Withdrawal threshold was determined as the filament at which the animal withdrew its paw at least two times out of five applications. To comprehensively present all data on response frequencies to graded von Frey hairs over all forces tested, we constructed a response frequency versus stimulus intensity (that is, von Frey force applied) curve per group for every time point tested. The integral of curve is represented as AUC^35^.

*Thermal sensitivity*. Paw withdrawal latency to a ramp of infrared heat was measured using a plantar test apparatus (Ugo Basile Inc.)^36^. The Cold Plate test was done with a Cold Plate (Bioseb) at 0 °C. The latency until the first withdrawal response of the hindpaw was recorded and mice were removed immediately. Cutoff latencies were set at 40 s to avoid tissue damage. For the two choice temperature preference assay, mice were placed in an apparatus that had plates at 30 °C and 18 °C (T2CT, Bioseb). The assay was initiated by placement on the 18 °C plate and the mouse position was tracked over 10 min.

Forelimb and hindlimb coordination and balance were measured using a rotarod apparatus (Accelerating Model, TSE). After a training and acclimatization period, mice were placed on the rotating rod and the time each animal could maintain its balance on the apparatus was recorded. The acceleration was from 1 to 30 r.p.m. over a 300-s period.

### Immunohistochemistry

Mice were perfused with 0.1 M phosphate buffer saline and 4% paraformaldehyde and the L3–L5 segment of the spinal cord was isolated and post-fixed for up to 16 h in 4% paraformaldehyde. Free-floating sections (50 μm, vibratome) or embedded cyrosections (12–25 μm) of mouse spinal cord were prepared using standard protocols. FluoroMyelin Red staining (1:300) was performed on 12 μm cyrosections according to the manufacturer’s protocol (Invitrogen). Unless elsewhere mentioned, immunohistochemistry was performed on 25 or 50 μm spinal cord sections with the following antibody dilutions in 7% normal horse serum in PBS: rabbit anti-ASPA: 1:1,000, polyclonal rabbit anti-MBP: 1:200 (Millipore), polyclonal rabbit anti-IBA-1: 1:1,000 (WAKO), monoclonal rat anti-CD68 (ED1 clone): 1:200 (Serotec), polyclonal rabbit anti-GFAP: 1:500 (DAKO), monoclonal mouse anti-Schwann cells (2E clone): 1:10,000 (Cosmo Bio Co.), polyclonal rabbit anti-β-TubulinIII: 1:800 (Sigma), monoclonal mouse anti-NeuN (A60 clone): 1:500 (Chemicon), monoclonal mouse anti-APP (A4 clone): 1:400 (Millipore), polyclonal rabbit anti-FOX3: 1:500 (EnCor Biotechnology), monoclonal mouse anti-APC (CC1 clone): 1:200 (Calbiochem), monoclonal mouse anti-Olig2 (211F1.1 clone): 1:500 (Millipore), monoclonal rat anti-CD3 (17A2 clone): 1:100 (BD Pharmingen), monoclonal rabbit anti-Ki67 (SP6 clone): 1:300 (Abcam). For Ki67 staining, antigen retrieval was adopted: tissue-embeded slides were quickly transferred into a staining jar filled with prewarmed 10 mM citrate buffer, pH 6; and incubated in water bath at 97 °C for 20 min. Slides were cooled till room temperature, rinsed with distilled water and further processed under normal staining protocol. Specimens were visualized using appropriate FITC/TRITC-conjugated secondary antibodies in 7% normal horse serum in PBS and confocal imaging was performed using a laser-scanning microscope (TCS SP2 AOBS, Leica) using similar illumination conditions for sections from diverse treatment groups.

### Cell counting

Immunohistochemistry was performed in 50 μm thick, vibratome sections of formalin-fixed spinal cord tissue of at least three animals from each of the experimental groups and time points. All cell counting analyses were carried out by an observer blind to the specific experimental conditions of the analysed tissues on images acquired at × 10 magnification. The number of cells positive for ASPA, IBA-1, CD68, GFAP and FOX3 as well as APP-positive axonal swellings was determined in three standardized, randomly placed fields of view of 100 μm^2^ each. Cell counts were done within the grey matter of the dorsal horn in spinal segments ranging from L3 to L5.

### RNA extraction and qRT–PCR

Either total spinal cord tissue or the L3–L5 segments were harvested and shock-frozen on dry ice. Total RNA was extracted using TRIzol RNA isolation reagent (Invitrogen Life Technologies) together with FastPrep Lysing Matrix D tubes (MP Biomedicals), pursuant to the manufacturer’s protocol. 1 μg of RNA was subsequently subscribed in cDNA by using SuperScript II Reverse Transcriptase from Invitrogen with random primers. One microlitre of an 1:5 dilution was used for quantitative real-time PCR using StepOnePlus Real-Time System (Applied Biosystems) with the QuantiTect SYBR Green RT-PCR Kit (Qiagen) in accordance with the manufacturer’s instructions. The thermal cycling conditions comprised 15 min at 95 °C, and 45 cycles of 15 s for denaturation at 95 °C and 30 s for annealing and extension at 60 °C. Primers for quantitative real-time PCR are obtained from Qiagen: Assay ID QT 00153888 for Myelin-associated glycoprotein (*mag*), QT 00096096 for proteolipid protein (*plp)*, QT00121044 for CD11b, QT00129787 for CD80, QT01055250 for CD86, QT01758407 for CD3. Expression is normalized to that of HPRT expression. Each qRT–PCR assay was performed in duplicates.

### Flow Cytometry

For flow cytomentric analysis of CNS-invading cells, mice were killed with CO_2_ and perfused intracardially with PBS. Spinal cord tissue was homogenized using MACS miltenyi C-Tubes and a gentleMACS dissociator according the manufacture’s guidelines and loaded on a 30:37:70% Percoll gradient. The 37:70% interphase was collected, the cells were washed and treated with Fc-block (1 μg per 10^6^ cells) before staining. Fluorescence staining was performed using the following antibodies purchased from BD or eBioscience: (CD11b, 1:1,000) eBioscience clone M1/70, (CD45.2, 1:200) eBioscience clone 104, (B220, 1:200) clone RA3-6B2, (CD4, 1:100) eBioscience clone GK1.5, (CD8, 1:300) BD clone 53-6.7. Analysis was performed on a FACSCantoII (BD Biosciences) and analysed by FlowJo software.

### Electron microscopy

Mice were anaesthetised and perfused transcardially with 0.9% NaCl, followed by fixative containing 4% formaldehyde, 2.5% glutaraldehyde (EM grade, Merck) and 0.5% NaCl in phosphate buffer, pH 7.4, according to the Karlsson and Schultz^37^ as described^38^. After dissection, pieces of lumbal spinal cord were postfixed with 2% OsO_4_ (Science Services) in 0.1 M phosphate buffer, pH 7.3, and embedded in EPON (Serva) after dehydration with ethanol and propylenoxide. Tissue in EPON-Block was then trimmed, using a Leica EM TRIM (Leica), to make area of interest, containing lateral spinothalamic tract, accessible. In the following ultrathin sections prepared with a Leica Ultracut S ultramicrotome (Leica), stained with an aqueous solution of 4% uranyl acetate followed by lead citrate^39^. EM pictures were taken with a Zeiss EM900 electron microscope (Zeiss) using a side-mounted 2 k CCD camera (TRS, Moorenweis, Germany). Axon-Analysis was performed using NIH ImageJ software (National Institutes of Health); a total area of 8 × 784=6,272 μm^2^ was analysed, counting axons on grid crossings. The grid device was set in ImageJ with an area per point of 2 square inch.

### Statistics

All data are expressed as mean±standard error of the mean (s.e.m.). Two-tailed unpaired Student’s *t*-test, analysis of variance for random measures with *post-hoc* Fisher’s test or repeated measures analysis of variance with *post-hoc* Fisher’s test were used. Changes with *P*<0.05 were considered to be significant.

## Author contributions

S.G., J.L., S.T., S.W., W.M., D.V., J.B., M.B. and T.R. performed the experiments and analysed data; D.V., S.T., S.W., K.K., A.W. and K.-A.N. provided important tools and conceptual input on the manuscript and helped to design experiments; R.K., S.G. and J.L. designed experiments and wrote the manuscript.

## Additional information

**How to cite this article:** Gritsch, S. *et al.* Oligodendrocyte ablation triggers central pain independently of innate or adaptive immune responses in mice. *Nat. Commun.* 5:5472 doi: 10.1038/ncomms6472 (2014).

## Supplementary Material

Supplementary Information Supplementary Figure 1.
