# Supplementary material for: Oligodendrocyte ablation triggers central pain independently of innate or adaptive immune responses in mice
Source: Nat Commun. 2014 Dec 1;5:5472. doi: 10.1038/ncomms6472 (PMC4268702; doi:10.1038/ncomms6472)
Supplement: Supplementary Information — Supplementary Figure 1. [file ncomms6472-s1.pdf]

Gritsch et al., supplementary information

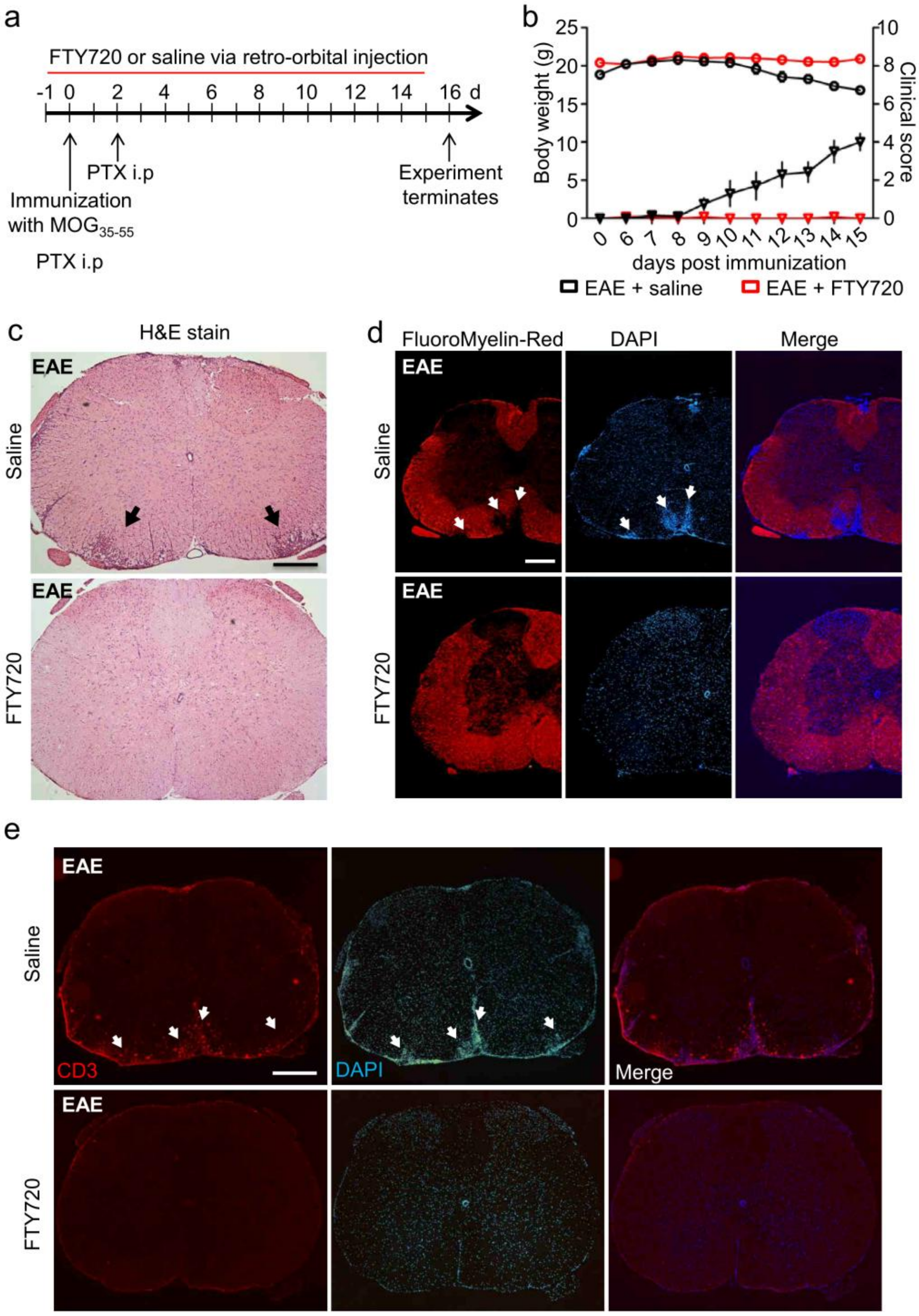

Supplementary Fig. 1: Use of MOG antigen-induced EAE model for establishing proof-of-principle for T-cell infiltration in the spinal cord, demyelination and the efficacy of FTY720.

a. Experiment scheme

b. EAE scoring on daily basis and body weight measurements

c. H&E staining on EAE spinal cord from EAE mice

d. Flouromyelin^{TM-}Red stained spinal cord from EAE mice showing demyelination in areas of immune cell infiltration (white arrows) and its blockade by FTY720.

e. Immunostaining with anti-CD3 to label infiltrating T-cells in EAE mice, which is blocked by treatment with FTY720.

Scale bars represent 300 microns in all panels.
